# Outcome of Septoplasty with Inferior Turbinectomy as an In-patient or Out-patient Procedure

**DOI:** 10.1038/s41598-019-44107-4

**Published:** 2019-05-20

**Authors:** Yung-Yuan Chen, Tzu-Chin Huang

**Affiliations:** 1 Department of Otorhinolaryngology, Cathay General Hospital, Taipei, Taiwan; 2 Department of Otorhinolaryngology, Hsinchu Cathay General Hospital, Hsinchu, Taiwan

**Keywords:** Prognosis, Outcomes research

## Abstract

The study aimed to compare the surgical outcomes of septoplasty with inferior turbinectomy between in-patient and out-patient groups. A total of 152 patients who underwent septoplasty with inferior turbinectomy between May 2012 and February 2013 were retrospectively reviewed and divided into in-patient group and out-patient group. The two groups were compared in three aspects: (i) consumption of medical resources, including National Health Insurance payments, patient surcharges, and total surgical expenses; (ii) prognostic indicators; and (iii) post-operative complications. There were no statistically significant differences between the prognostic indicators or post-operative complications in the 2 study groups except for “duration of nasal decongestant use”. The in-patient group had higher medical resource consumption in all categories. In conclusion, septoplasty with inferior turbinectomy can be performed cost-effectively as an out-patient procedure with satisfactory quality and adequate safety.

## Introduction

Nasal septal deviation (NSD) occurs in 77–90% of the general population^1,2^. Septoplasty with inferior turbinectomy is one of the most common surgeries for NSD and chronic hypertrophic rhinitis (CHR). The major goals of this operation include improving nasal flow, relieving obstructive sleep apnea, and rhinogenic headache. Although there are a number of possible approaches to conduct a septoplasty, most surgeons utilize a similar technique that includes submucosal resection of the cartilaginous nasal septum (SMR), controlled osteotomy of bony nasal septum, and concomitant inferior turbinectomy. This kind of surgery can be performed as either an ambulatory or an in-patient procedure.

Previous studies have revealed the variation in the day-surgery rate among septoplasties in different parts of the world. In 1998, it was estimated that only 2.2% of all septoplasties were performed as day cases in the United Kingdom (UK)^3^. In 2005, a 250-bed hospital in Italy had 69% of their septoplasties performed as ambulatory procedures^4^. A more recent report from Portugal demonstrated that 77% of the septoplasties were undertaken as outpatient procedures^5^. In Taiwan however, most septoplasties with concomitant inferior turbinectomy are still performed as in-patient procedures. The increasing trend towards day-surgery procedures due to continuous improvements in medical practices as well as economic factors has profoundly changed the management of patient hospitalization^6–8^.

Under the hypothesis that septoplasty with inferior turbinectomy would be a good candidate for day-case surgery without compromising patient safety or clinical outcome, this retrospective study is to compare the prognosis, complications, and expense associated with this surgery as an in-patient or out-patient procedure.

## Methods

One hundred and fifty-two patients who underwent septoplasty and inferior turbinectomy at Hsinchu Cathay General Hospital, Hsinchu, Taiwan between May 2012 and February 2013 were included in this study. Every patient had nasal septal deviation and inferior turbinate hypertrophy confirmed by physical examination. A standardized pre-operative questionnaire including age, sex, and other systemic diseases was collected from every patient. Patients with major systemic co-morbidity (e.g., uremia, liver cirrhosis, or cancer) were excluded. Patients who underwent concomitant endoscopic sinus surgery or rhinoplasty (both cosmetic and functional) were also excluded from the analyses.

Preoperatively, the patients could decide whether to admit for the procedure or not. The out-patient group left the hospital almost immediately after the surgery following a brief 30-minute break in the observation room. The in-patient group were admitted to the hospital several hours before the surgery and stayed in the wards for one or two nights after the procedure (mostly one night).

All of the procedures were performed by a single surgeon (senior author) under local anesthesia using the same operative technique. Prior to the SMR surgery, lidocaine with 1:100000 adrenaline was injected to the septal mucosa on both sides. A Killian incision was made and bilateral mucoperichondrium and mucoperiosteum were elevated from the cartilaginous and bony septum. The deviated septal cartilage and bone were removed by forceps under endoscope, while preserving adequate caudal and dorsal strut. At the end of the surgery, the mucosa was closed at the incision line using one or two absorbable trans-mucosal sutures, together with potassium titanyl phosphate (KTP) laser vaporization of both inferior turbinates. As to the nasal packing, non-resolvable Merocel® (standard 8 cm, expandable polyvinyl acetate; Metronic Xomed, Jacksonville, FL) or biodegradable Nasopore (Forte plus 8 cm, Polyganics, Rozenburglaan, Groningen, The Netherlands) coated with neomycin ointment were used for nasal packing depending on the preference of the patient (we didn’t have preference from the medical professional point of view). For patients who received Merocel, the packing was removed within 24–48 hours of the operation. For patients who received Nasopore, the packing was left in the nasal cavity where it could break down spontaneously without any manipulation. Any residual fragments and crust were cleaned out during the follow-up visit (usually at day 7 post-surgery).

The post-operative regimens for the patients in the two groups were quite similar, including one-day oral antibiotic therapy (cephalexin 500 mg qid), together with topical nasal steroids. Analgesics were not routinely given. Decongestant medication was given whenever patients complained of nasal obstruction, which made it an ideal indicator for post-operative nasal airway patency. All of the patients were regularly followed-up in the clinic, and the post-operative complications were recorded in the medical charts. The end-point of follow-up was smooth nasal breathing post-operatively with no residual crust or exudates found in either nasal cavity. The better the wound healed, the shorter recovery time and the fewer decongestant medications the patient needed.

A retrospective review of medical records and data collection were conducted by an independent reviewer. Several parameters were investigated following the surgical procedure, and differences between the two study groups were compared in three aspects: (i) consumption of medical resources, including National Health Insurance payments (US dollars), patient surcharges (US dollars), and total surgical expenses (sum of the previous two items); (ii) prognostic indicators, including the duration of nasal decongestant use, number of follow-up visits, and total period to complete recovery; and (iii) post-operative complications, including post-operative hemorrhage, subsequent septal hematoma, septal perforation, nasal adhesions (synechiae), and wound infection. Post-operative hemorrhage was defined as massive bleeding that required emergency management or re-packing at any time during the follow-up period.

### Statistical analysis

Statistical analyses were performed using SPSS for Windows version 16.0 (SPSS Inc., Chicago, IL). Categorical variables were analyzed using the chi-square test and Fisher’s exact test, as appropriate. Differences between the groups were analyzed using the t-test. Statistical significance was set at *p* < 0.05.

### Ethics approval and consent to participate

The hospital Institutional Review Board (Hsinchu Cathay General Hospital, Hsinchu, Taiwan) approved this study. All experiments were performed in accordance with the Hsinchu Cathay General Hospital Research Ethics Committee guidelines and regulations. Informed consent was obtained from all the patients.

## Results

### Characteristics of the study subjects

The study cohort of 152 patients had a mean age of 37.0 ± 11.2 years (range, 18–65 years) and included 99 men (65.1%) and 53 women (34.9%). The two groups (in-patient and out-patient groups) were not significantly different in terms of age, gender, and packing material (shown in Table 1).

**Table 1:** Clinical Characteristics of the Study Subjects^a^.

	In-patient (n = 104)	Out-patient (n = 48)	*p*
Age, mean ± SD, year	36.1 ± 11.1	39.1 ± 11.8	0.438*
**Gender**			0.680**
Male	67	32	
Female	37	16	
**Packing**			0.056**
Merocel	59	35	
Nasopore	45	13	

^a^Data are presented as number of patients unless otherwise indicated.

*t-test.

**Chi-square tests.

### Comparison of prognostic indicators

The period to complete recovery and the number of follow-up visit showed no statistical difference between in-patient and out-patient groups (*p* = 0.928 and 0.579 respectively). Only the duration of nasal decongestant use was significantly different in in-patient and out-patient groups (shown in Table 2).

**Table 2:** Prognostic Indicators.

	In-patient (n = 104)	Out-patient (n = 48)	*p*
Period required for complete recovery, mean ± SD, week	4.2 ± 1.6	4.2 ± 1.5	0.928
Number of follow-up visit, mean ± SD	3.3 ± 1.1	3.2 ± 1.1	0.579
Duration of nasal decongestant use, mean ± SD, week	1.9 ± 0.4	1.4 ± 0.5	<0.001

Note: By t-test.

### Incidence of post-operative complications

In terms of post-operative complications, none of them showed statistical difference between in-patient and out-patient groups, and there was no case of wound infection in either group (shown in Table 3).

**Table 3:** Incidence of Post-Operative Complications^a^.

	In-patient (n = 104)	Out-patient (n = 48)	*p*
Post-operative hemorrhage	3 (2.9%)	0 (0%)	0.552
Septal hematoma	2 (1.9%)	1 (2.1%)	1.000
Septal perforation	0 (0%)	1 (2.1%)	0.316
Nasal adhesions (synechiae)	2 (1.9%)	2 (4.2%)	0.591
Wound infection	0 (0%)	0 (0%)	—

^a^Data are presented as number (percentage) of patients.

Note: By Fisher’s exact test.

### Analysis according to nasal packing materials

To eliminate the influence of nasal packing, we analyzed the patients separately according to their nasal packing materials (Merocel or Nasopore). In patients using Merocel as nasal packing, we found no differences between in-patient and out-patient on prognostic indicators or post-operative complications except for the parameter “duration of nasal decongestant use”. In patients using Nasopore as nasal packing, we found similar result (shown in Tables 4 and 5).

**Table 4:** Prognostic Indicators (According to Different Nasal Packings).

	Merocel (n = 94)			Nasopore (n = 58)		
	Out-patients (n = 35)	In-patients (n = 59)	*p*	Out-patient (n = 13)	In-patient (n = 45)	*p*
Period required for complete recovery, mean ± SD, week	4.1 ± 1.5	4 ± 1.7	0.740	4.3 ± 1.7	4.4 ± 1.6	0.790
Number of follow-up visit, mean ± SD	3 ± 1.1	3.2 ± 1.2	0.576	3.6 ± 1.1	3.5 ± 1	0.656
Duration of nasal decongestant use, mean ± SD, week	1.4 ± 0.5	1.8 ± 0.4	<0.001	1.6 ± 0.5	2 ± 0.4	0.018

Note: By t-test.

**Table 5:** Incidence of Post-Operative Complications (According to Different Nasal Packings)^a^.

	Merocel (n = 94)			Nasopore (n = 58)		
	Out-patients (n = 35)	In-patients (n = 59)	*p*	Out-patients (n = 13)	In-patients (n = 45)	*p*
Post-operative hemorrhage	0 (0%)	0 (0%)	—	0 (0%)	3 (6.7%)	0.339
Septal hematoma	1 (2.9%)	1 (1.7%)	0.706	0 (0%)	1 (2.2%)	0.588
Septal perforation	1 (2.9%)	0 (0%)	0.192	0 (0%)	0 (0%)	—
Nasal adhesions (synechiae)	1 (2.9%)	2 (3.4%)	0.887	1 (7.7%)	0 (0%)	0.061
Wound infection	0 (0%)	0 (0%)	—	0 (0%)	0 (0%)	—

^a^Data are presented as number (percentage) of patients.

Note: By Fisher’s exact test.

### Comparison of medical resource consumption

In comparing the medical expenses between the study groups, in-patient group had higher medical resource consumption across all categories than out-patient group. (shown in Table 6).

**Table 6:** Consumption of Medical Resources.

	In-patients (n = 104)	Out-patients (n = 48)	*p*
Patient surcharge (mean ± SD, US dollars)	256.67 ± 149.88	117.96 ± 107.49	<0.001
National Health Insurance payment (mean ± SD, US dollars)	625.07 ± 32.23	506.13 ± 1.23	<0.001
Total surgical expense (mean ± SD, US dollars)	881.73 ± 161.89	624.10 ± 107.65	<0.001

Note: By t-test.

## Discussion

The National Health Insurance (NHI) system was established in 1995 in Taiwan to cover most of the medical and health service demands of the population. Similar to the National Health Service in the UK however, increasing pressures on NHI resources has led to reassessment of many common procedures with a view to establish the feasibility and safety of performing these as day cases. The increased in-patient load also makes it difficult to guarantee beds for elective patients^8–10^.

Within the last decade, there has been a rapid expansion in ambulatory surgery with day operations already accounting for half of the surgical workload in the US and the UK. However, there are widely differing perceptions about the appropriateness of day-surgery for septoplasty. Septoplasty is being performed routinely as a day-procedure in many countries in the world, but in Taiwan, the majority of such surgeries are still in-patient procedures, with the patient staying overnight at the hospital until the nasal packing is removed the following day^8,11–14^. The emerging perspectives on septoplasty as a routine outpatient procedure are now under review in Taiwan, yet few studies have been performed with local data.

We found no statistically significant difference between in-patient and out-patient groups in the analyzed prognostic indicators such as the period to complete recovery and the number of follow-up visit, but the duration of nasal decongestant use. This means the two groups reached the recovery end-point at the same time but more medications were prescribed in the in-patient group. We speculated that the reason might lie on different patient behaviors in and out of the hospital. For in-patient group, decongestant prescription could be easily accessed in the ward whenever they complained of nasal obstruction, which was the most frequent complaint immediately after nasal surgery. As a result, in-patients tended to be more dependent on the decongestant medication, even after they went home. In contrast, the use of decongestant medication was significantly reduced in out-patient group during follow-up period.

Some surgeons may have concerns that an out-patient procedure without continuous nursing care may increase the risk of post-operative infection or other complications. In our study, the incidence of post-operative complications (i.e., post-operative hemorrhage, subsequent septal hematoma, septal perforation, or nasal adhesions) showed no significant difference between in-patient and out-patient groups. In addition, no wound infections were recorded in either of the study groups. During literature review, we found no previous report of more complications associated with out-patient septoplasties compared with in-patient ones.

To control for the possible influence of variant nasal packing on our current results, we seperated the patients according to the nasal packing materials (Merocel or Nasopore), and the analysis between in-patient and out-patient showed similar results as previously mentioned. Consistently, a review of the literature also indicated no difference in post-operative complications from the use of different packing materials^15,16^.

For medical resources consumption, our present analysis has revealed that the out-patient approach is less costly and places a lower demand on medical resources. We calculated that the total surgical expenses for the out-patient group were about one-third lower than the in-patient cases. Previous studies in other countries also showed that the shift from in-patient to out-patient procedure in performing the same surgery equates to an overall savings of 41–50% to the provider, which is consistent with our current findings^7,8,17^.

It is noteworthy that the guidelines for day-case surgery from the Royal College of Surgeons in the UK indicate that the unexpected admission rate should be less than 3%^6^. In our present study series, only one of the patients scheduled for an ambulatory procedure asked to be admitted immediately after the surgery due to anxiety and discomfort. This led to an unexpected admission rate of 2.0% (1/48), which is thus within the aforementioned guidelines. Other than this patient (unexpected change of mind on admission), we did not have any re-admission after surgery in our patient group.

Our study does have some limitations. First, owing to the retrospective nature of our present analyses, the study patients were not randomly allocated into the in-patient and out-patient groups. The patients’ preferences, general condition, or other personal factors (e.g., socioeconomic status, occupation, and insurance) that can impact the decisions regarding hospital admission may have impacted on our current findings. Second, the severities of nasal septal deviation and turbinate hypertrophy varied between patients. Despite our efforts to standardize the surgical procedures and post-operative regimens, the individual differences could still affect the prognosis and post-operative complications. Third, we didn’t have an objective measurement for post-operative nasal airway patency e.g., acoustic rhinometry or rhinomanometry. Therefore, we took “the duration of nasal decongestant use” as the prognosis indicator instead.

## Conclusions

Our current study findings indicate that septoplasty with inferior turbinectomy is a good candidate for an out-patient procedure not only for the cost-effectiveness but also for the surgical quality and safety, as evidenced by the comparable prognostic indicators and post-operative complications between the in-patient and out-patient approaches.

## Data Availability

The datasets generated and analyzed during the current study are not publicly available due to hospital policy but are available from the corresponding author on reasonable request.
